# Retraction of: Circular RNA NF1-419 enhances autophagy to ameliorate senile dementia by binding Dynamin-1 and Adaptor protein 2 B1 in AD-like mice

**DOI:** 10.18632/aging.206282

**Published:** 2025-07-31

**Authors:** Chen Diling, Guo Yinrui, Qi Longkai, Tang Xiaocui, Liu Yadi, Yang Xin, Hu Guoyan, Shuai Ou, Yong Tianqiao, Wang Dongdong, Xie Yizhen, Burton B. Yang, Wu Qingping

**Affiliations:** 1 State Key Laboratory of Applied Microbiology Southern China, Guangdong Provincial Key Laboratory of Microbial Culture Collection and Application, Guangdong Open Laboratory of Applied Microbiology, Guangdong Institute of Microbiology, Guangdong Academy of Sciences, Guangzhou 510070, China; 2 Research and Development Institute of Chinese Materia Medica, Guangdong Pharmaceutical University, Guangzhou 510006, China; 3 Department of Pharmacy, The Fifth Affiliated Hospital of Guangzhou Medical University, Guangzhou 510700, China; 4 Sunnybrook Research Institute, Department of Laboratory Medicine and Pathobiology, University of Toronto, Toronto, Canada

**Keywords:** circular RNA, aging, astrocyte, biological function, autophagy

**This article has been retracted:** “Aging” has completed its investigation of this paper. Following communication with the authors we found that two corresponding authors, Dr. Qingping Wu and Dr. Burton Yang, were not involved in the preparation, revision, and proofreading of this paper. The first author, Dr. Chen Diling, confirmed he listed them as corresponding authors without their permission. Additionally, Dr. Diling noted unintentional duplication of two sets of Western blot bands (Erk1/2 and Beclin-1) in Figure 4, and two p53 IHC images in Supplementary Figure 6. These unresolved concerns bring into question the validity of the reported results and the article's adherence to “Aging”'s authorship policy. Therefore, the Aging Editors have decided to retract this article. All authors were informed of this decision and agreed with it.

